# Adjunctive use of laser biostimulation with nonsurgical periodontal therapy: a split-mouth, randomized, case-control study in diabetic and nondiabetic periodontitis patients

**DOI:** 10.55730/1300-0144.5797

**Published:** 2023-11-18

**Authors:** Funda AKANSEL, Umur SAKALLIOĞLU, Müge LÜTFİOĞLU, Feyza OTAN ÖZDEN, Adil KARADAĞ

**Affiliations:** 1 Department of Periodontology, Faculty of Dentistry, Ondokuz Mayıs University, Samsun, Turkiye; 2 Private Practice, London, United Kingdom; 3 Department of Medical Microbiology, Faculty of Medicine, Ondokuz Mayıs University, Samsun, Turkiye; 4 Private Practice, Samsun, Turkiye

**Keywords:** Laser biostimulation, photobiomodulation, periodontal treatment, therapy, periodontitis, diabetes mellitus, IL-1β, IL-10

## Abstract

**Background/aim:**

Laser biostimulation therapy (LBT) is suggested to have positive effects on periodontal healing. This study evaluated LBT with nonsurgical periodontal therapy (NSPT) in diabetes mellitus (DM) and systemic health (SH) conditions.

**Materials and methods:**

Thirty periodontitis patients (15 with DM and 15 with SH) were included in the study, which had a split-mouth design, by applying LBT in the mouth of the same systemic condition. Thus, 4 study groups were formed, as 1) NSPT − DM: NSPT alone in DM, 2) NSPT + LBT − DM: NSPT + LBT application in DM, 3) NSPT − SH: NSPT alone in SH, and 4) NSPT + LBT − SH: NSPT + LBT application in SH. NSPT was performed on days 15, 30, 37, 44, 51, 58, and 65. LBT was performed 6 times on days 30, 37, 44, 51, 58, and 65 with an Nd:YAG laser. The plaque index (PI), gingival index (GI), bleeding on probing (BOP), probing pocket depth (PPD), and clinical attachment level (CAL) were assessed as the clinical parameters and recorded at baseline and days 30, 37, and 72. Gingival crevicular fluid levels of interleukin 1 beta (IL-1β) and IL-10 were evaluated by ELISA as the biochemical parameters at baseline and on days 30, 37, and 72.

**Results:**

Clinical parameters had improved in all of the groups on day 72 (p < 0.01). PPD and CAL improved more in the DM group with NSPT and LBT group than in the DM group with NSPT without LBT on day 37 (p < 0.05). IL-1β decreased and IL-10 increased in all of the groups on day 72 (p < 0.01). This change was more evident in the DM group with NSPT and LBT than in the DM group with NSPT without LBT on day 7 (p < 0.05).

**Conclusion:**

These results revealed the short-term impacts of LBT on periodontal healing, which return to ineffectiveness with repeated irradiation. Therefore, it may be speculated that LBT via the protocol herein may have a short-term antiinflammatory contribution to NSPT, only in impaired healing conditions such as DM.

## 1. Introduction

Nonsurgical periodontal therapy (NSPT) is the fundamental procedure of the periodontal treatment process for the management of periodontal disease, i.e. to reduce periodontal inflammation, reduce periodontal pocket, and regain periodontal attachment loss [1]. However, adjunctive protocols have been suggested to increase the efficacy of this process, particularly for modulating the healing response. To date, the one that stands out among these protocols is dental laser application which has been concluded to be an adjunctive or an alternative treatment approach to standard periodontal treatments [2,3]. Laser biostimulation therapy (LBT) is recommended due to its photochemical roles in enhancing tissue growth and regeneration, resolving inflammation, reducing pain, and promoting wound healing [2]. LBT has been investigated by means of its antiinflammatory and healing-promotion actions in periodontium with conflicting outcomes, i.e. some have reported positive effects while others have suggested ineffectiveness [4–6]. The biostimulative effect of laser on tissue healing has drawn significant attention, not only in healthy individuals but also in patients with systemic problems causing impaired wound healing such as diabetes mellitus (DM) [3,7,8]. Patients with poor metabolic control frequently present impaired wound healing and LBT utilization as a treatment method for diabetic ulcers have shown positive healing effects [9]. However, limited data exist about the adjunctive benefits of LBT on periodontal healing or periodontal inflammatory response in patients with DM.

Inflammation is the basic periodontal tissue response in both the destruction and healing process, and the convenient tissue response is important in creating adequate healing. Impaired inflammation in DM specifically affects the outcomes of periodontal wound healing. It was shown that expression of local inflammatory factors in gingival crevicular fluid (GCF) increases in the periodontitis patients with poorly controlled DM [10,11], which is a reflection of accelerated tissue injury in the diseased periodontium [3]. While periodontal homeostasis is maintained via proinflammatory [i.e. interleukin 1 beta (IL-1β)] and antiinflammatory (i.e. IL-10) cytokine balance, any destabilization beneficial for the proinflammatory cytokines results in periodontal disease, tissue destruction, and/or impaired healing [12,13]. Therefore, wound healing in DM may require an adjunctive approach during periodontal therapy, particularly to biostimulate the healing periodontal tissues.

Thus, the present research aimed to test whether LBT would aid in improving the outcomes of NSPT, comparing this treatment process in DM and systemic health (SH) conditions.

## 2. Materials and methods

This study was designed as a split-mouth, randomized, and case-controlled clinical trial. It was approved by the Local Medical Ethics Committee of Ondokuz Mayıs University (No: OMU KAEK 2012/49) and Drug and Medical Device Agency of Turkish Ministry of Health (No: 761317e). Informed consent was obtained from all of the study participants in accordance with the Declaration of Helsinki (1975, revised in 2002). The study was registered at ClinicalTrials.gov (Registration number: NCT04253613).

### 2.1. Study group allocation

The research population comprised male and female (35–55 years of age) periodontitis patients who neither smoked nor consumed alcohol. Inclusion criteria were: 1) having been diagnosed as uncontrolled DM (via the American Diabetes Association criteria, with ≥7 HbA1c level) [14] at least 2 years before the study or having a SH condition; 2) having no other systemic problems or conditions or not undergoing any other drug usage/regimen; and 3) having been diagnosed with Grade III, Stage C periodontitis (GI ≥ 2, BOP (+), PPD ≥ 6 and CAL ≥ 5 mm) [15] and not having prior periodontal treatment for at least for 6 months. Exclusion criteria were as follows: 1) having any systemic disease other than DM known to affect periodontal status; 2) being a smoker; 3) having undergone antibiotic therapy in the previous 3 months; and 4) having had surgical or NSPT in the previous 6 months.

Thus, 4 study groups were constituted as: 1) NSPT − DM: NSPT alone in DM, 2) NSPT + LBT − DM: NSPT + LBT application in DM, 3) NSPT − SH: NSPT alone in SH, and 4) NSPT + LBT − SH: NSPT + LBT application in SH. This group allocation was performed as a split-mouth design regarding the patients’ systemic conditions (SH or DM) and LBT application. Therefore, patients allowing at least 2 symmetrical same-group teeth (incisor or premolar) in the same mandible for NSPT and NSPT + LBT were selected from the periodontitis patients.

### 2.2. Clinical examination and sampling

The clinical periodontal status was assessed using the Silness and Löe plaque index (PI) [16], Löe and Silness gingival index (GI) [17], bleeding on probing (BOP), probing pocket depth (PPD), clinical attachment level (CAL), and radiographical examination. An automated periodontal probe (Florida Probe, version FP 32/7.2.2, Florida Probe Corp., Gainesville, USA) calibrated in mm was used to measure the PPD and CAL at 6 sites on each tooth (mesio-buccal, mid-buccal, disto-buccal, mesio-lingual, mid-lingual, and disto-lingual locations). A William’s probe (Hu-Friedy, Chicago, IL, USA) was utilized for measurement of the PI, GI, and BOP at the same sites. For each tooth, the highest periodontal examination value sites of all of the clinical parameters were identified, but only the sites concomitantly demonstrating bone resorption in standard panoramic radiographs were recorded for the following measurements, GCF sampling and data analyses.

GCF was obtained for the biochemical assessments and this collection was performed in the morning. Sampling sites were first isolated with cotton rolls, saliva was removed, and supragingival plaque (if any) was taken using a sterile curette. A filter paper strip (Periopaper, OraFlow, Inc., Amityville, New York, USA) was placed into the gingival crevice where mild resistance was felt and left there for 30 s. Strips contaminated with saliva or blood were discarded and the sampling was repeated. The GCF volume in each strip was determined using an electronic impedance device (Periotron 8000, ProFlow, Inc., Amityville, New York, USA), and the stripes were then stored in sterile 400-μL Eppendorf tubes (Eppendorf AG, Hamburg, Germany) at −60 °C until the biochemical analyses. All of these procedures were performed by the same researcher (FA).

### 2.3. NSPT and LBT

NSPT conventionally included oral hygiene instruction/reinforcement, supra-sub/gingival scaling, root-planing, and dental polishing. All of the supra-gingival procedures were done with hand or ultrasonic/rotary instruments, whereas the subgingival ones were done using hand instruments only. No systemic or local medications were utilized during the NSPT process. Patient compliance, oral hygiene, and periodontal statuses were checked, and the procedures were repeated in the required sites during the further NSPT sessions on days 15, 30, 37, 44, 51, 58, and 65.

LBT was applied as an adjunctive procedure for NSPT. In each mouth, the tooth/site selection for LBT usage was randomly determined by coin toss (left or right) and the one symmetrical to it served as the placebo site (where the inactive laser device was used in the non-LBT groups) just holding the laser device for the same time, period, and manner as in the LBT groups. LBT was performed 6 times on days 30, 37, 44, 51, 58, and 65 using a 1064 nm Nd:YAG laser device (Fotona AT, Fidelis Plus III, Ljubljana, Slovenia). The sites received 5 s of laser application using the hand piece with noncontact (at a distance of 1 cm from the related gingiva) at a frequency of 10 H, in mini short pulse mode, 0.25 W of mean-power, 1.25 J of energy, interaction area of 0.28 cm^2^, energy density of 4 J/cm^2^, and power density of 0.8 W/cm^2^ [18]. All of these procedures were performed by the same researcher (FA).

### 2.4. Study protocol and flow chart

The study protocol and flow chart are demonstrated in the Figure. Briefly, all of the clinical measurements were performed before NSPT (baseline) and on days 30, 37, and 72, whereas GCF samples were taken 24 h following the periodontitis diagnosis but before NSPT (baseline) and on days 30, 37, and 72. NSPT was initiated following the periodontitis diagnosis and first GCF collection (baseline), which was continued with the described NSPT and LBT processes.

### 2.5. Biochemical analyses

IL-1β and IL-10 level alterations in the GCF throughout the study were determined for the biochemical assessments.

GCF was first isolated from the stored paper stripes by a given protocol [19]. A total of 150 μL of 2% bovine serum albumin in phosphate-buffered saline (0.01 M, pH: 7–7.2) was added to the stripe-containing tubes and they were incubated at 4 °C for 60 min. Following incubation, a sterile drill was used to bore a hole in the bottom of each tube, which was placed into another 1500-μL microcentrifuge tube. These nested tubes were first exposed to a vortex procedure for 30 s and then centrifuged at 10,000 *g* and 4 °C for 5 min. Thus, 200 μL of GCF was obtained from each tube, repeating this procedure, and it was equally utilized (as 100 μL) for the IL-1β and IL-10 calculations.

The IL-1β and IL-10 levels were determined by enzyme-linked immunosorbent assay (ELISA) using ELISA kits (Human IL-1β ELISA kit/catalog no: EK0392 and Human IL-10 ELISA kit/catalog no: EK0416, Boster Biological Technology Co. Ltd., California, USA) according to the manufacturer’s instructions. Reactions were terminated by adding an acid solution and color-changes were measured spectrophotometrically at 450 nm. IL-1β and IL-10 were identified by the standard curve values, calculating the concentrations via ELISA dilution coefficients, which were precorrected for the GCF volumes. Results were expressed both as pg/μL and pg/30 s. All of these laboratory analyses were performed by the same researcher (AK).

### 2.6. Statistical analyses

The required sample size was calculated by a statistical software program (Minitab 17, Minitab LLC, Pennsylvania, USA) regarding the CAL levels. Statistical analyses were performed using IBM SPSS Statistics for Windows 22.0 (IBM Corp., Armonk, NY, USA) and the results were presented as the mean ± standard deviation or median (minimum–maximum). The Shapiro–Wilk test was performed to test the normality of the groups. Comparisons within the groups were evaluated by nonparametric Friedman and Wilcoxon signed rank (with Bonferroni correction) tests, and parametric ANOVA with the post hoc Tukey test. Intergroup comparisons were evaluated via parametric Student’s t and nonparametric Mann–Whitney U tests.

## 3. Results

A minimum of 15 patients per group were identified to ensure an accuracy of α = 0.05 at a confidence interval of 82%. Thus, 30 periodontitis patients (15 DM and 15 SH) were included in the split-mouth design. There were no differences in terms of age (mean: 45.4 ± 7.24-DM and 43.9 ± 7.85-SH) or sex (7/8-DM and 8/7-SH) between the DM and SH groups (p > 0.05). All of the participants completed the NSPT or NSPT+LBT and follow-up period without missing any appointments. Healing was uneventful in all of the patients and no adverse reactions (e.g., burning sensation, pain) related to laser irradiation were reported.

### 3.1. Clinical assessments

Clinical measurements and calculations are given in Table. All of the parameters showed statistically significant improvements after baseline on days 30, 37, and 72 in all of the groups. The PPD and CAL in the NSPT+LBT-DM showed better improvement compared the NSPT-DM on day 37 (p < 0.01). There were no other differences for the clinical data in terms of time-dependent paired comparisons of LBT in the DM groups (p > 0.05). The clinical alterations did not vary for the time-dependent paired comparisons of LBT between the NSPT-SH and NSPT+LBT-SH groups (p > 0.05).

### 3.2. Biochemical assessments

Biochemical measurements and calculations are given in the Table. IL-1β reduction and IL-10 elevation were observed during all of the examination periods in the DM groups, respectively (p < 0.01). These alterations were higher in the NSPT+LBT-DM group than in the NSPT-DM group on day 37 (p < 0.01). There were no other differences in the biochemical data in terms of time-dependent paired comparisons of LBT in the DM groups (p > 0.05). IL-1β decreased and IL-10 increased gradually in all of the SH groups during all of the examination periods regardless of LBT (p < 0.01) without any differences for the time-dependent paired comparisons of LBT (p > 0.05).

## 4. Discussion

It has been reported that LBT may positively affect the healing process, such as biomodulation, normalizing blood vessel permeability, and boosting the microcirculation by causing vasodilation [2]. However, findings about the interaction between LBT and healing are still limited and controversial. According to the present study, LBT administered with an Nd:YAG-laser and by the given noncontact protocol adjunctive to NSPT may have a shorter- or earlier-term positive impact on periodontal healing.

The GI and BOP scores herein demonstrated a significant gradual reduction in both the DM and SH groups at each examination time-point regardless of LBT. Regarding the time-dependent paired comparisons of LBT, no significant differences were observed for the GI and BOP between the DM and SH groups during the examination periods. Similarly, significant improvements were detected in the PPD and CAL in the DM and SH groups throughout the study period regardless of LBT. Unlike the SH groups, in which there were no differences in the time-dependent paired comparisons at any of the examination times, the PPD and CAL improved significantly in the NSPT+LBT-DM group compared to the NSPT-DM group on day 37 (after the first LBT). However, this alteration was not detected between the NSPT+LBT-DM group and NSPT-DM group on the other examination days. Several studies have revealed that the clinical effects of adjunctive LBT with an Nd:YAG laser were similar to those obtained with NSPT alone [20–22], whereas it was also reported to be more efficient than NSPT alone [23–28]. Again, studies of different laser types and application methods have reported that LBT did not provide any additional clinical benefits over conventional treatment by means of reducing the PPD and CAL measurements [4,5,20,28], as well as suggesting better outcomes with LBT [6,26,27,30]. LBT with an Nd:YAG laser, which is applied with contact and as a single dose adjunctively, has been suggested to be more effective in terms of positive PPD outcomes when evaluated for follow-up periods of 3, 6, 9, and 12 months [23,25–27,30]. Gómez et al., in an LBT study with contact form an Nd:YAG laser, concluded that the healing response of tissue for up to an 8-week period may be shorter to obtain the outcomes of LBT [20]. However, Castro des Santos et al. did not find any significant effects of NSPT + LBT with contact using a diode laser on the BOP scores after 3, 6, and 12 months in both DM and SH patients [11]. Furthermore, regardless of the application technique, there are various reports indicating that diode laser applied in addition to periodontal treatment in DM results in significant improvements in GI scores, cellular parameters, and BOP scores after 1-month [33], 3-month [34], and 6-month [34] follow-ups. Regarding the PPD and CAL, adjunctive effects of LBT with several laser types caused significant improvements in DM patients after 1, 3, and 6 months [3,34,35]. Conversely, no additional benefit of LBT was obtained based on the PPD and/or CAL in DM patients with diode laser applied with noncontact [33] and contact [11] adjunctive to NSPT. In the current study, a 6-week follow-up period was performed in order to evaluate the periodontal healing process. There are limited studies that have evaluated LBT with an Nd:YAG laser during NSPT in DM and SH patients for shorter periods (<8 weeks). In a split-mouth designed study [35], periodontitis patients (with DM or SH) with a PPD of ≥4 mm received NSPT+LBT with an Nd:YAG laser, which was applied with contact and in a single session. The clinical parameters were seen to have better improvement with LBT in both the DM and SH patients after 4 weeks without any differences at the 12-week examination. The abovementioned clinical data, together with the clinical findings herein, suggest that the heterogeneity of research designs, dental laser types and their settings, and application protocols may alter the study outcomes and may evoke controversies that may complicate proper comparisons and/or conclusions [36,37].

It has been concluded that any discrepancy in the IL-1β/IL-10 ratio of the GCF is related to the course and process of the periodontal destructive state [13]. In the present study, IL-1β decreased and IL-10 increased during the evaluation periods at both the LBT (+) and LBT (–) sites. Regardless of the LBT application, the findings were consistent with previous studies that have detected IL-1β and IL-10 alterations after NSPT [12,38]. On the other hand, rather few studies have focused on the LBT and GCF interaction and have evaluated the effect of LBT with an Nd:YAG laser on the GCF contents of DM patients adjunctive to NSPT. Qadri et al. [6] and Makhlouf et al. [30] applied multiple LBT sessions with diode laser and they observed greater decreases in the GCF volume [6] and PPD [6,30] of laser treated groups without any significant differences in IL-1β at 1 week after the last laser irradiation. Calderin et al. [5] compared single (1 day after NSPT) and recurrent doses (1, 2, 4, 7, and 11 days after NSPT) of LBT with a diode laser and compared the IL-1β levels after 4 and 8 weeks. They reported significant decreases in both laser groups compared to NSPT alone, but the decrease in IL-1β was more significant in the laser group receiving repeated doses. In regard to LBT with an Nd:YAG laser, it has been reported that GCF IL-1β levels significantly decrease compared to NSPT alone after different follow up periods, i.e. 1 week [27], 1 month [20,27], 3 months [24], or 6 months [24]. Nevertheless, it has also been reported that NSPT+LBT with an Nd:YAG laser may not create a significant decrease in IL-1β comparatively [23]. In the present study, there was no significant impact of laser on the IL-1β level at any time-dependent paired comparison in the SH group and this finding was in agreement with several studies that did not suggest any IL-1β differences with LBT regardless of the laser type [6,23,30].

There are limited studies that have evaluated the GCF IL-10 levels by means of the effect of NSPT+ LBT with an Nd:YAG laser on periodontal disease management. In one study on periodontitis (aggressive periodontitis according to the previous periodontitis classification) the patients’ clinical, microbiological, and inflammatory mediator changes were evaluated at 15, 30, 60, 180, and 365 days after performing LBT with a diode laser in a single session with contact [13]. They revealed that NSPT+LBT reduced IL-1β on days 15 and 30, and increased IL-10 on days 30 and 60. The findings herein did not demonstrate any significant alterations in the IL-10 levels due to LBT application in the SH patients. An in vitro study with cultured human gingival fibroblasts in high-glucose medium determined the antiinflammatory effect of low-level laser irradiation by assessing the expression of proinflammatory genes including IL-1β with quantitative real-time polymerase chain reaction, and LBT was suggested as a beneficial tool for the treatment of periodontal disease in DM patients [43]. However, inadequate findings are currently available for LBT with an Nd:YAG laser in this regard. In the current study, the time-dependent paired comparison of NSPT with NSPT+LBT revealed a decrease in IL-1β and increase in IL-10in conjunction with PPD and CAL improvements in the DM group on day 37. LBT utilization has been suggested for activating target cells to improve normal wound healing and to induce sufficient healing in patients with deficient healing dynamics such as those with DM. Normalization is the keystone of LBT, and it aims to restore homeostasis, and therefore, LBT may assist the natural healing process when an abnormal condition is encountered in the wound, i.e. impaired healing dynamics, excessive cell/tissue damage, and even a more painful wound. Thus, the resultant clinical benefits such as pain relief, improved tissue repair and increased blood circulation may normalize the healing [44]. In this sense, the day-37 findings herein were in agreement with studies claiming an unremarkable impact of LBT in SH patients [5,32,33,45], and the remarkable impact of LBT on healing improvement in compromised wounds [7,8], such as in DM.

It may be speculated that any possible limited influence of LBT may be attributed to the altered reactions of tissues to laser irradiation due to their different characteristics. Not only the laser wave-lengths but also the cell types in the wound/granulation tissue have been found to be of importance when evaluating the biomodulatory efficacy of laser [40,41]. When laser is absorbed less by a target tissue/cell, more laser energy is transmitted beyond the target and this outcome will be detected as ineffectiveness of the application. Different penetration depths may also influence both the clinical and laboratory studies and they may explain the variabilities of the study results better [29]. Various wave-lengths (including Nd:YAG) and the cell type influence the cellular response to laser irradiation by means of cellular proliferation and secretory action [29,40]. Again, the healing phase and laser application time/frequency (i.e. on the first day with NSPT or following NSPT) are rather important because of the expected cellular activity alterations in the wound after LBT [37,42].

Some researchers have suggested several sessions of LBT to achieve positive outcomes following conventional periodontal therapy [5]. Conversely, it has been claimed that low-power laser application doses are cumulative, and thus, several sessions during a shorter period may lead to inhibitory-level actions [43–46]. Miscellaneous data about this phenomenon could not reveal a consensus about the optimal application protocol [5,6,31,33,34,39]. However, it is widely accepted that the wavelength and application time, together with the number of applications, are essential factors for the cellular stimulation and expected LBT effects [2,46–48]. Consistently, when the LBT dose reaches the maximum value that is needed for cellular activation, any doses beyond this value may evoke diminished or unseen responses, which may even produce negative effects [2]. Karu [48] suggested a window-specificity at certain wavelengths and energy densities at which the positive effects of LBT can be expected.

Given the current lack of clarity on this subject, more well-designed prospective and case-control clinical trials are needed to obtain adequate information about the application method, dosage, and proper type of laser that will be the most suitable for obtaining the possible positive effects of LBT adjunctive to periodontal treatment. In this regard, it may be speculated that: 1) the study design and one type of laser protocol utilization and 2) the selection of a small number of systemic conditions may be the main limitations of the current study. Therefore, further research comparing different study designs and laser protocols should be carried out to obtain more perceptible study outcomes.

## 5. Conclusion

The findings of this study indicated that LBT administered with an Nd:YAG laser in addition to NSPT may have a short-term antiinflammatory effect, particularly in DM, which becomes ineffective with repeated irradiation.

## Figures and Tables

**Figure:**
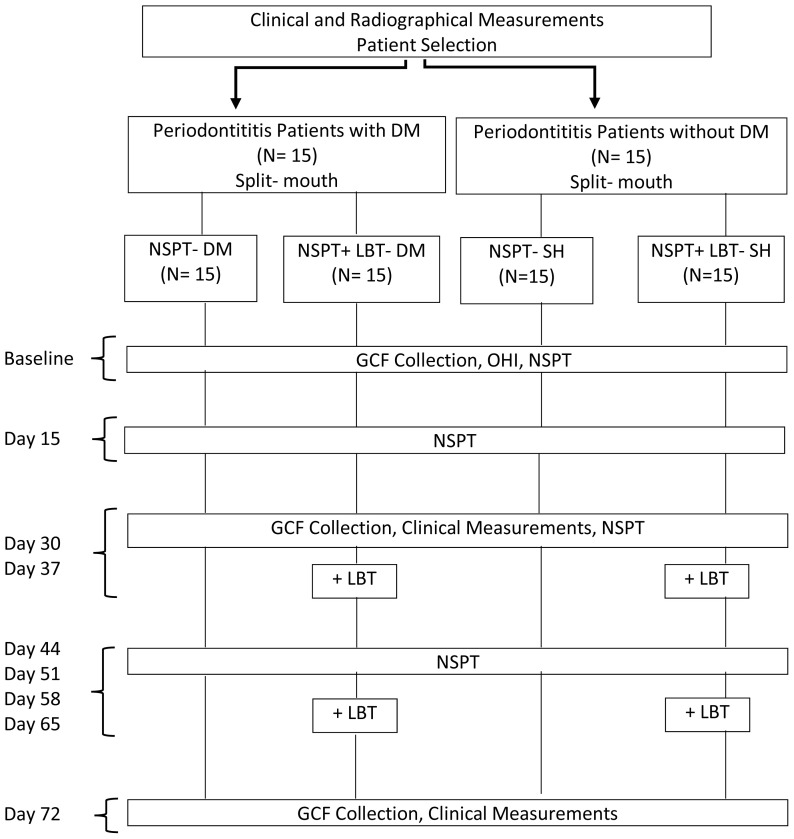
Flow-chart of the study is shown in the diagram. DM: diabetes mellitus, SH: systemic health; NSPT: nonsurgical periodontal therapy, LBT: laser biostimulation therapy, GCF: gingival crevicular fluid, OHI: oral hygiene instructions.

**Table:** Time-dependent clinical and biochemical findings [mean ± SEM, median (minimum-maximum)] in the study groups.

		NSPT-DM (n = 15)				NSPT-SH (n = 15)			
Clinical parameters		Baseline	Day 30	Day 37	Day 72	Baseline	Day 30	Day 37	Day 72
PPD (mm)	LBT (−)	7.01 ± 1.04	6.43 ± 1.04 *	6.15 ± 1.04¥,*,**	4.01 ± 1.04*,***	5.50 ± 1.03	4.99 ± 0.92§	4.82 ± 0.46§,§§	2.50 ± 1.04§,§§§
	LBT (+)	6.73 ± 0.47	6.15 ± 0.47 *	5.32 ± 0.47¥,*,**	3.73 ± 0.47*,***	5.91 ± 0.33	5.33 ± 0.33§	4.88 ± 0.32§,§§	2.91 ± 0.33§,§§§
CAL (mm)	LBT (−)	8.51 ± 0.71	7.66 ± 0.71*	7.31 ± 0.71¥,*,**	5.51 ± 0.71*,***	7.23 ± 0.71	6.29 ± 0.25§	5.93 ± 0.62§,§§	4.23 ± 0.43§,§§§
	LBT (+)	8.56 ± 0.61	7.71 ± 0.61*	6.65 ± 0.61¥,*,**	5.56 ± 0.61*,***	7.30 ± 0.43	6.45 ± 0.31§	5.73 ± 0.51§,§§	4.30 ± 0.31§,§§§
PI	LBT (−)	3 (2–3)	1 (1–2)*	2 (2–3)*	1 (1–2)*	2 (1–2)	1 (0–1)§	1 (1–2)§	0 (0–1)§
	LBT (+)	3 (2–3)	1 (0–2)*	2 (1–3)*	1 (0–2)*	2 (1–2)	0 (0–1)§	0 (0–1)§	1 (0–2)§
GI	LBT (−)	2 (1–3)	1 (0–2)*	1 (0–2)*	0 (0–1)*	2 (1–2)	1 (0–1)§	1 (0–1)§	0 (0–1)§
	LBT (+)	2 (1–3)	1 (0–2)*	1 (0–2)*	0 (0–1)*	1 (0–3)	0 (0–2)§	0 (0–2)§	1 (0–2)§
BOP	LBT (−)	1 (1–1)	0 (0–1)*	0 (0–1)*	0 (0–0)*	1 (1–1)	1 (0–1)§	0 (0–1)§	0 (0–0)§
	LBT (+)	1 (1–1)	0 (0–1)*	0 (0–1)*	0 (0–1)*	1 (1–1)	0 (0–1)§	0 (0–1)§	0 (0–1)§
Biochemical parameters									
IL-1β (pg/μL)	LBT (−)	826.46 ± 61.57	275.38 ± 6.50*	209.80 ± 18.81¥,*,**	183.90 ± 7.60*,***	588.08 ± 116.96	258.45 ± 12.96§	202.24 ± 16.07§,§§	164.56 ± 04.96§,§§§
	LBT (+)	818.14 ± 63.52	271.98 ± 12.94*	185.56 ± 12.13¥,*,**	183.79 ± 4.62*, ***	587.54 ± 105.63	258.14 ± 8.75§	196.58 ± 9.61§,§§	166.45 ± 6.93§,§§§
IL-1β (pg/30 s)	LBT (−)	781.25 ± 81.61	120.88 ± 7.97*	81.49 ± 6.36¥,*,**	60.05 ± 2.22*,***	479.22 ± 80.94¥	96.92 ± 6.68§	78.30 ± 6.60§,§§	60.05 ± 2.22§,§§§
	LBT (+)	755.63 ± 51.42	118.64 ± 11.51*	72.38 ± 9.17¥,*,**	59.85 ± 0.69*,***	474.11 ± 70.94	100.76 ± 19.40§	77.62 ± 4.25§,§§	59.85 ± 0.69§,§§§
IL-10 (pg/μL)	LBT (−)	41.80 ± 4.93	126.16 ± 10.54 *, **	203.89 ± 34.15¥,*,**	811.37 ± 87.06*,***	52.52 ± 5.35	173.55 ± 20.14§	269.66 ± 58.45§,§§	1290.8 ± 234.8§,§§§
	LBT (+)	43.4 ± 2.59*, **	132.19 ± 26.68	252.96 ± 40.31¥,*,**	824.16 ± 88.41*,***	52.76 ± 7.64¥	174.55 ± 37.90§	260.14 ± 35.11§§	1274.1 ± 151.9¥, §, §§
IL-10 (pg/30 s)	LBT (−)	39.18 ± 1.61	55.14 ± 2.49 *	78.83 ± 9.92¥, *, **	265.0 ± 29.09 *, ***	42.8 ± 0.94¥	64.24 ± 2.81§	102.89 ± 14.86§§	391.64 ± 70.86¥
	LBT (+)	40.11 ± 2.33 *	57.07 ± 10.78 *	97.15 ± 5.87¥, *, **	268.81 ± 41.1*,***	42.39 ± 0.70¥, *	65.66 ± 2.7§	102.53 ± 12.75¥, *	385.16 ± 45.68*

¥ Significant difference between LBT (−) and LBT (+) at the same sampling periods (Student’s t and Mann–Whitney U tests, p < 0.01).

* significant difference from baseline in DM (paired sample-t test, p < 0.001 for PPD, CAL, IL-1β, and IL-10, Friedman test + Wilcoxon test with Bonferonni adjustment, p < 0.008 for PI, GI, and BOP).

** Significant difference between days 30 and 37 in DM (paired sample-t test, p < 0.001 for PPD, CAL, IL-1β, and IL-10, Friedman test + Wilcoxon test with Bonferonni adjustment, p < 0.008 for PI, GI, and BOP).

*** Significant difference between days 37 and 72 in DM (paired sample-t test, p < 0.001 for PPD, CAL, IL-1β, and IL-10, Friedman test + Wilcoxon test with Bonferonni adjustment, p < 0.008 for PI, GI, and BOP).

§ Significant difference from the baseline in SH (paired sample-t test, p < 0.001 for PPD, CAL, IL-1β, and IL-10, Friedman test + Wilcoxon test with Bonferonni adjustment, p < 0.008 for PI, GI, and BOP).

§§ Significant difference between days 30 and 37 in SH (paired sample-t test, p < 0.001 for PPD, CAL, IL-1β, and IL-10, Friedman test + Wilcoxon test with Bonferonni adjustment, p < 0.008 for PI, GI, and BOP).

§§§ Significant difference between days 37 and 72 in SH (paired sample-t test, p < 0.001 for PPD, CAL, IL-1β, and IL-10, Friedman test + Wilcoxon test with Bonferonni adjustment, p < 0.008 for PI, GI, and BOP).
